# Quantifying the area at risk using the infarct lateral border: importance of infarct transmurality

**DOI:** 10.1186/1532-429X-14-S1-O24

**Published:** 2012-02-01

**Authors:** Christoph J Jensen, Lowie M Van Assche, Han W Kim, Lubna Bhatti, Peter Filev, Ki-Young Kim, Michele Parker, Igor Klem, Raymond J Kim

**Affiliations:** 1 Duke Cardiovascular MR Center, Duke University Medical Center, Durham, NC, USA

## Background

The wavefront phenomenon describes the transmural progression of myocardial infarction (MI) from endocardium to epicardium with increasing ischemia duration. A corollary is once subendocardial MI has developed, the infarct lateral border (InfarctLatBor) delineates the Area-at-risk (AAR) lateral border, and thus, can be used to measure overall AAR size. However, with short ischemia time a confluent subendocardial layer of infarction may not develop, and InfarctLatBor may underestimate AAR size. The transmural extent of infarction necessary for InfarctLatBor to accurately reflect AAR size is unknown.

In-vivo assessment of InfarctLatBor with delayed-enhancement-CMR (DE-CMR) has been compared with surrogates of the AAR (ECG, angiographic scores, T2-weighted-CMR). However, no comparison exists with a pathology-based truth standard of the AAR (i.e microspheres). We sought to examine: (1) on pathology studies, the threshold of infarct transmurality necessary for the InfarctLatBor to accurately delineate the AAR, and (2) the ability of in-vivo DE-CMR (via InfarctlatBor assessment) to quantify the AAR in comparison with pathology.

## Methods

In 15 canines, MI with various infarct transmuralities was produced by temporary occlusion (50-120mins) of the LAD or LCx. A complete LV short-axis stack (7mm thickness, no gap) of DE-CMR images were obtained following gadoversetamide administration (0.2mmol/kg). Prior to sacrifice, the infarct-related-artery was reoccluded at the same site (same suture) and microspheres (1-10μ, 2 million, Duke scientific corp.) were injected into the left atrium to determine AAR size (AAR_PATH_). After TTC-staining the infarct lateral border was used to estimate AAR size (InfarctLabBor_PATH_).

## Results

**Figure 1:**
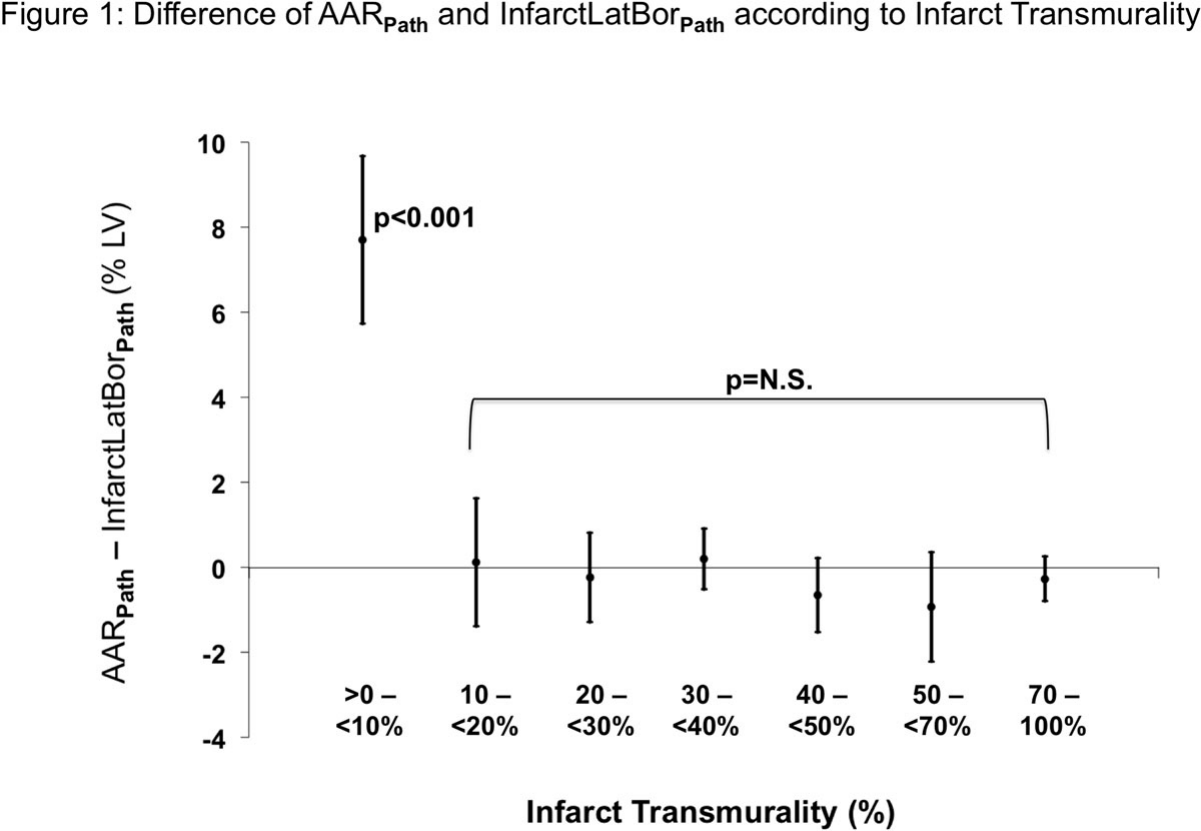
The infarct lateral border ((InfarctLatBor**_Path_**) is used to estimate true area-at-risk size delineated by microspheres (AAR**_Path_**). Note, underestimation of AAR size by the infarct lateral border occurs only in slices with mean infarct transmurality <10% (p<0.001). In groups with higher transmurality no bias was found (p=N.S.).

Comparing pathology-based measurements per-slice (N=114), InfarctLatBor_PATH_ slightly underestimated AAR_PATH_ (28.2±25.9% vs. 28.9±25.4%, bias -0.6±2.9%, p=0.03), though correlation was excellent (r=0.994). In slices with mean infarct transmurality <10% InfarctlatBor_PATH_ underestimated AAR_PATH_, whereas no systematic under- or overestimation occurred when infarct transmurality was >10% (**Figure**1). Similarly, on a per-heart basis, in-vivo InfarctlatBor_DE-CMR_ slightly underestimated AAR_PATH_ (25.2±13.3% vs. 26.8±12.4%, bias -1.6±2.5%, p=0.03), and again the correlation was excellent (r=0.979). The greatest underestimation (-8.4%ofLV) was found in the subject with lowest mean infarct transmurality (11%) and highest number of slices (N=4) with infarct transmurality <10%. Excluding this subject, the maximum bias was lower than -4%ofLV for all other subjects.

## Conclusions

The lateral border of infarction allows for precise quantification of true AAR size unless a subendocardial layer of infarction less than 10% transmural is present. In-vivo DE-CMR assessment of the infarct lateral border can be used to accurately estimate AAR size, however, underestimation may occur if mean infarct transmurality is near 10%.

## Funding

This study was funded in part by following NIH-grant: 5R01HL064726-07.

